# An evaluation of University of Cape Town medical students’ community placements in South Africa

**DOI:** 10.4102/phcfm.v4i1.448

**Published:** 2012-11-09

**Authors:** Claudia S. Naidu, Virginia Zweigenthal, James Irlam, Leslie London, Johannah Keikelame

**Affiliations:** 1 Primary Health Care Directorate, University of Cape Town, South Africa; 2 Department of Public Health and Family Medicine, University of Cape Town, South Africa

## Abstract

**Background:**

Fourth-year medical students at the University of Cape Town (UCT) work closely with stakeholders in community teaching sites to conduct community-based research projects and follow-up health promotion interventions during their Public Health training.

**Objectives:**

This study evaluated the placements as a learning experience from the perspectives of past students and community stakeholders.

**Methods:**

A total of 32 projects were randomly selected out of 232 projects undertaken during 2006, 2008 and 2009. Two students and a stakeholder involved with each project were sampled. A standardised survey was emailed to students and in-depth interviews were held with stakeholders.

**Results:**

Fifty two per cent of 64 students and 57% of 25 stakeholders responded. Most students felt that the placements enhanced their academic experience and confidence in research skills, and were an effective form of learning. Perceived challenges included time constraints and, for a minority, inadequately prepared settings and stakeholders. Stakeholders felt that the placements empowered the communities and prepared students for the realities of working as a medical professional. They viewed students as a valuable resource and believed that student projects addressed important community myths and health problems. Recommendations from students and stakeholders included more time for the Public Health block, follow-up interventions for greater continuity, and better alignment of projects with stakeholder programmes.

**Conclusion:**

The evaluation reveals both the importance and challenges of community placements and identifies areas of improvement. Despite the limited duration of the placements, they offered valuable community-based learning experiences for the students and worthwhile benefits for the communities.

## Abstrait

**Une évaluation des formations communautaires des étudiants en médecine de l'Université du Cap en Afrique du Sud**

**Contexte:**

Les étudiants en médecine de quatrième année de l'Université du Cap (UC) travaillent en étroite collaboration avec des parties prenantes des sites d'apprentissage communautaires afin de réaliser des projets de recherche communautaires et d'effectuer un suivi des interventions de promotion de la santé pendant leur formation en santé publique.

**Objectifs:**

Cette étude a évalué ces formations en tant qu'expériences d'apprentissage du point de vue d'anciens étudiants et des parties prenantes de la communauté.

**Méthodes:**

Au total, 32 projets ont été sélectionnés au hasard sur les 232 projets entrepris en 2006, 2008 et 2009. Un échantillon de deux étudiants et une partie prenante ont été sélectionnés pour chaque projet. Une étude standardisée a été envoyée aux étudiants par email et des entretiens approfondis ont été réalisés avec les parties prenantes.

**Résultats:**

Cinquante-deux pour cent des 64 étudiants et 57% des 25 parties prenantes ont répondu à l’étude. La plupart des étudiants considéraient que les stages avait enrichi leur expérience universitaire et amélioré leur confiance en leurs compétences de recherche, et que ceci constituait une forme d'apprentissage efficace. Les défis perçus incluaient les contraintes de temps et, pour une minorité, des environnements et des parties prenantes préparées de manière inadéquate. Les parties prenantes considéraient que les formations apportaient beaucoup aux communautés et préparaient les étudiants aux réalités de l'exercice de la profession médicale. Elles voyaient les étudiants comme des ressources précieuses et considéraient que les projets des étudiants permettaient de traiter des mythes et des problèmes de santé communautaires importants. Les étudiaient comme les parties prenantes recommandaient de consacrer davantage de temps au bloc d'apprentissage qu'est la santé publique, de réaliser des interventions de suivi afin d'assurer une meilleure continuité et de mieux aligner les projets aux programmes des parties prenantes.

**Conclusion:**

L’évaluation révèle à la fois l'importance et les défis associés aux formations communautaires et identifier les points à améliorer. En dépit de la durée limitée de ces formations, elles permettaient d'offrir des expériences d'apprentissage communautaires précieuses aux étudiants et des avantages non négligeables aux communautés.

## Introduction

The training of an appropriately skilled and sustainable workforce has been acknowledged as a critical strategy for the improvement and development of a health care system that is fair, equitable, responsive and accountable.^1^ The report of the Global Commission on Education of Health Professionals for the 21st century calls for a new era of health professional education which promotes quality, embraces teamwork, upholds a strong service ethic, and is centred around the interests of patients and populations.^2^ Graduates should be equipped to assess and respond to community needs in relation to service provision; understand the genetic, environmental and psychosocial causes of illness and diseases; and apply primary health care principles. The incorporation of public health as a ‘science of preventing disease, promoting health and prolonging life’^3^ into the curriculum is an important platform from which to develop many of the attributes required of ‘tomorrow's doctors’.^4^ Placements within communities offer practical learning experiences where students and community stakeholders can apply principles central to improving population health.^5^

The World Health Organisation^6^ has identified community-based education (CBE) as an effective method of training health personnel who are responsive to community needs. Placement of students in communities is an opportunity to engage in ‘educational practice that links formal academic learning with the process of addressing real-world, community-identified needs’.^7^ CBE enables students to better recognise, understand, support and engage in collective action to address identified community health needs,^8^ and prepares them for their future working environments as primary healthcare practitioners. Studies have shown that community-based education experiences motivate students to practice in community health care settings and improve attitudes towards rural and general practice.^9–11^ Long-term follow-up research suggests that undergraduate exposure to primary care and community settings lead to career preferences that translate into socially responsive practice decisions.^12^ Graduates of community-based and socially responsive programmes were more likely to choose primary care residencies, to show more positive attitudes towards a rural career, and also to have developed appropriate attitudes and interpersonal skills for primary health care.^13, 14^

Another feature of this form of learning paradigm is the potential for reciprocity between students and the community in which they serve and learn. A 2007 National Institute of Clinical Excellence review in the United Kingdom of the evidence for effectiveness of community-engaged initiatives suggested that the majority of ‘engaged’ individuals perceive improvements in physical and psychological health; valuable psychosocial benefits including establishing social relationships, supporting self-empowerment; and building trust and understanding between communities.^15^ However, relatively few attempts have been made to explore the impact that community engagement activities have on the individuals and communities involved.

## Background

### CBE in the University of Cape Town Faculty of Health Sciences

CBE plays an important role in promoting the primary health care approach of the University of Cape Town (UCT) Faculty of Health Sciences.^16^ Key principles of this approach include promoting equity in health care; health promotion; intersectoral collaboration; and community involvement.^17^ The Faculty envisions CBE as a learning environment which is community-based, adequately resourced, caring, and physically secure.^18^ CBE can help strengthen the district health system through the provision of on-site teaching and service delivery, and promote mutually beneficial relationships between the community, service providers, and the Faculty of Health Sciences.^16, 18–20^ CBE thus aims to address the health needs of groups beyond hospital settings, and brings students closer to communities which they may service in the future.

In their fourth year of study students complete an eight-week rotation in Public Health and Health Promotion across five community-based teaching sites. The curriculum covers epidemiology, evidence-based practice, occupational and environmental health, understanding and working in health systems, research methods, behaviour change and community organisation theories as well as principles of health promotion, health communication and community participation. The rotation is structured around lectures, seminars, group work, field visits and community placements. Site facilitators are appointed for each community and are responsible for facilitating liaison between community stakeholders and students, and provide feedback to students, stakeholders and course coordinators.

During the first four weeks students working in groups of four to five research a prioritised health problem within a specified community site. Research findings are presented to relevant stakeholders, and a feasible health promotion project based on the results of their research is developed in consultation with stakeholders. In the remaining weeks students learn to apply the fundamental principles of the Ottawa Charter for Health Promotion,^21^ namely enablement, advocacy and mediation as well as action, learning, planning and reflection cycles to plan, design and implement their health promotion projects. Through practical engagement on site, students learn and apply skills used in health promotion such as networking, advocacy, communication, organising, facilitation, planning and negotiation. In preparing students for population-oriented practice in South Africa, the course therefore emphasises experiential learning and critical self-reflection, team work, community participation and empowerment.^22^

Importantly, student placements at community sites should be of mutual benefit to students and the academic institution, as well as to the community, local health services and organisations. Regular assessment is required to test this assumption, and to ensure that learning objectives are being met.

We therefore undertook this study to evaluate the fourth-year MBChB Public Health community placements as a learning experience from the perspectives of the students and community stakeholders. The objectives were to assess the gain in knowledge and skills reported by students from the community placement and related projects; their views on the value of the projects; their views on the benefits and challenges of their Public Health rotation; and the views of stakeholders on the value of or difficulties with students’ work in communities.

## Methods

### Study population

The study population comprised medical students who completed their fourth-year Public Health rotation and stakeholders who had hosted students during their rotation.

### Sampling

Projects were randomly selected from all Public Health projects completed during 2006, 2008 and 2009. Two students and a stakeholder involved with each project were then sampled.

### Data collection

A questionnaire was designed, which comprised closed multiple-choice, rating and agreement scale questions, as well as verbatim open- ended questions. The questionnaires were emailed to current and past students along with a consent form and description of the project and research aims. An interview guide probing perceptions of the benefits and difficulties associated with the projects was used for stakeholder interviews. Stakeholder interviews lasted on average 20 minutes, and were recorded and transcribed with their consent.

### Data analysis

Survey data were entered into EPIDATA and analysed using STATA (version 10) software. Interview transcripts were reviewed and segments of data were inductively sorted into categories representing emerging themes and related sub-themes. The development of themes was not based on frequency but on ‘substantive significance’, that is, the consistency of themes across and within study participants.^23^ Themes were examined, refined and merged through an ongoing and iterative analysis process. Qualitative data analysis software, ATLAS.ti, was used to manage and organise the data for the coding of themes, allowing flexibility and improved validity in the data analysis process.^24^

Ethical approval of the study was obtained from the Ethics Committee of the Faculty of Health Sciences of the University of Cape Town.

## Results

A total of 32 projects out of 232 projects undertaken during the years 2006, 2008 and 2009 were selected. Project topics ranged from management of chronic diseases to stress, nutrition, and sexual behaviour (Box 1). They were undertaken in primary care clinics, schools, non-governmental organisations, police services, community health centres, and households in Khayelitsha, Langa or Vanguard, and Atlantis or Mamre, and in a partner non-governmental organisation (Cancer Association of South Africa). Most were cross-sectional studies investigating the knowledge, attitudes, behaviour and practices regarding an issue that stakeholders considered important. The research was followed by a consultative process of developing a feasible and relevant health promotion intervention that related to the findings.

BOX 1 Community based placements – project topics and settings.

**Healt topics†**

Child Health e.g. immunisation, nutrition and diarrhoea

Chronic diseases e.g. hypertension, diabetes

Cancer awareness and screening practices

Reproductive and sexual health e.g. contraception, HIV and AIDS

Mental health issues e.g. stress, substance abuse

Health of older people

**Settings**

Schools

Schools

Community health centres

Communities

Non-government organisations

Police Services

†, Research projects led to health promotion interventions on these topics based on findings.

### Student survey

A response rate of 52% (*n* = 33) was achieved from the 64 students sampled; 28 students returned electronic questionnaires which are analysed below, and five gave general telephonic feedback on their placement experiences. All students had favourable perspectives on the placements, with many saying that they were ‘valuable’ (36%; *n* = 10); provided a learning opportunity (29%; *n* = 8); provided practical experience (21%; *n* = 6); and enhanced knowledge (18%; *n* = 5). As one commented:

‘Really awesome experience. I really feel that because my placement was so well designed and we had such motivated facilitators … I really learnt a lot about myself and about how public health actually works…’ (Fifth-year student)

#### Development of research and health promotion skills

**FIGURE 1:**
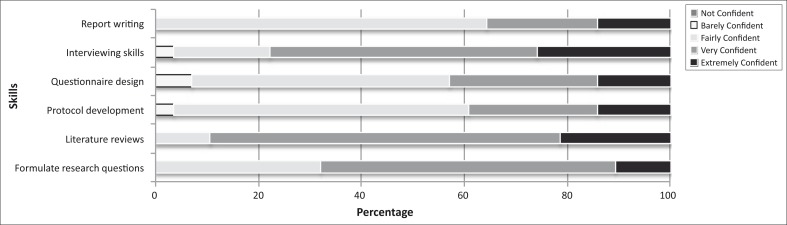
Student confidence in research skills

A majority of students felt very or extremely confident in formulating a clear research question (68%; *n* = 19); in finding and reviewing the literature (89%; *n* = 25); and in conducting interviews (78%; *n* = 21). Most felt fairly confident in writing a research protocol (57%; *n* = 16); designing a valid questionnaire (50%; *n* = 14); and in writing up a clear and coherent research report (64%; *n* = 18) (Figure 1).

The majority of students felt fairly to extremely confident in using health promotion theories and principles to develop interventions to address identified health problems (89%; *n* = 25), and considered the research project an excellent opportunity to learn about health problems in the community (71%; *n* = 20), and an effective form of learning and applying their epidemiology and research methods skills (68%; *n* = 19).

Students felt that the community-based health promotion activities were fairly to extremely useful in providing an understanding of health rights (93%; *n* = 26) and ethics (93%; *n* = 26) in primary care settings; understanding the role of community stakeholders (86%; *n* = 24) and the importance of establishing collaborative partnerships (82%; *n* = 23); developing their skills as reflective practitioners (93%; *n* = 26), and working effectively in a team (93%, *n* = 26).

#### Value of community-based placements

**FIGURE 2:**
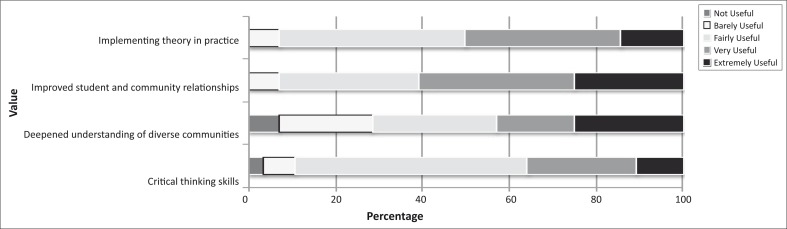
Student assessment of the value of the community-based placements.

A majority of the students found the community placements to be fairly to extremely useful in helping them to work better with people of different backgrounds (61%; *n* = 17) and in connecting course content to community-based activities (50%; *n* = 14). Fifty-four per cent (*n* = 15) and 36% (*n* = 10) respectively, however, considered the placement barely or fairly useful at enhancing their critical thinking skills. A minority (29%; *n* = 8) did not find the placement useful or found it barely useful in helping them to better understand cultures, races, ethnicities and socio-economic classes different from their own (Figure 2).

‘I was able to use theoretical knowledge that I had gained, in a practical setting. It was particularly valuable in strengthening my research and interpretation skills.’ (Fifth-year student)

Over a third of students (36%; *n* = 10) believed that their fieldwork, research and health promotion recommendations were well utilised by the stakeholders, but 39% (*n* = 11) felt unsure. The remaining 25% (*n* = 7) thought that they were not well utilised. Most students (68%; *n* = 19) felt that their groups’ efforts were valued by the stakeholders, whilst 21% (*n* = 6) felt unsure.

#### Benefits and challenges

**FIGURE 3:**
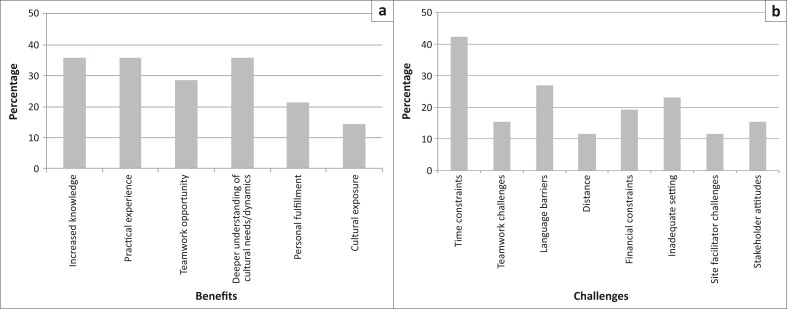
Perceived benefits and challenges of community-based placements, as cited by students. (a) Perceived benefits to students (%). (b) Perceived challenges faced by students (%).

Over a third of students (36%; *n* = 10) felt that they had benefitted in all three areas: increased knowledge; a deeper understanding of cultural needs and/or dynamics; and practical experience. Twenty nine per cent (*n* = 8) reported benefitting mainly from the opportunity to work as part of a team, and 21% (*n* = 6) felt personally fulfilled by their community placement experiences (Figure 3).

‘I felt that it was an excellent learning experience and should be continued in the years to follow as it has helped me to better myself in being a future health professional.’ (Fifth-year student)

The main challenges reported in implementing the research project and health promotion intervention included time limitations (42%; *n* = 11); communication barriers (27%; *n* = 7); logistical issues or inadequate settings (21%; *n* = 6); financial constraints (18%; *n* = 5); a poor relationship with stakeholders (14%; *n* = 4); teamwork challenges (14%; *n* = 4); site facilitator challenges (11%; *n* = 3); and long travelling distances (11%; *n* = 3) (Figure 3).

#### Student recommendations

Fourteen per cent of students (*n* = 4) recommended more time be allocated to the Public Health block, 14% suggested follow-up studies on previous student projects, and 54% (*n* = 15) offered a variety of suggestions, including smaller groups, fewer lectures, more biostatistics revision classes, and supervisors with higher levels of competence.

### Stakeholder interviews

#### Value of student placements

As stakeholders were often responsible for multiple projects, the sample of 32 projects yielded 25 stakeholders, 13 of whom (52%) were available for interviews. Stakeholders believed that the value of the placements was to encourage students to enter the public sector and empower and make communities feel cared for. They also felt that they prepare and expose students to the realities of working as a medical professional in contexts where they may practice in future. A majority of stakeholders reported that their expectations were well managed by both the students and UCT, and that the students’ feedback to them was adequate and useful. Feedback sessions were considered important as they offered the opportunity for stakeholders to comment on the process, outcomes, and the way forward, as well as for students to inform stakeholders of challenges faced. One stakeholder emphasised the need for improved communication and collaboration between the organisation and the university.

#### Benefits and challenges of student placements

All stakeholders perceived a benefit from the students being placed in community settings and working on community health problems. Almost half of the stakeholders viewed students as a valuable resource, and a third considered the placements an educational experience for stakeholders. One considered the health promotion activities as a valuable educational contribution for the community. Many also felt that the placements and students’ projects identified and addressed important community health issues, addressed popular myths, assessed knowledge and attitudes of the community, and provided meaningful, relevant and accessible information and health promotion tools to stakeholders. Students initiated new and reinforced partnerships between stakeholders and other local organisations, which was beneficial in developing successful health promotion interventions and creating networks for future collaboration.

Most stakeholders believed challenges accompanying the student placements to be minimal. The few challenges mentioned related to limited space to accommodate student activities, time limitations, and communication barriers. As one commented:

‘…They brought so many skills that are different from the time I qualified, we never used to focus on the community. So it really was important from an educational experience for us.’ (Stakeholder respondent 2)

#### Stakeholder recommendations

A few stakeholders recommended that student topics be determined in accordance with the organisations’ health promotion targets, as this would maximise the potential to successfully sustain interventions in future. Many stakeholders felt there should be greater continuity with previous projects in order to sustain the benefits, reinforce community knowledge, and potentially reach a greater number and variety of people. One commented that:

‘It would be beneficial if they did follow up and did a new campaign, because they'll be dealing with different people and they would be able to reinforce what they did.’(Stakeholder respondent 3)

Other recommendations included greater student involvement on a clinical level, greater collaboration between UCT and stakeholders, longer duration of placements, greater opportunities for more formal student volunteering beyond the block period, and having at least one student who could speak the common language of the community.

## Discussion

UCT's curricular learning outcomes include the ability to work reflectively within teams and apply theoretical knowledge and skills in a community healthcare setting; to display an understanding of research ethics, health rights, and health promotion; and to acknowledge the role of community stakeholders in primary care contexts and the importance of partnering with local community health services.

The majority of students expressed positive views of the community placements, and considered them to be an effective and valuable form of learning. Experiences of working with the community, conducting research, and implementing health promotion interventions were considered fairly to extremely important in achieving most of the learning outcomes. These findings concur with the World Health Organisation's primary rationale for CBE in preparing students ‘for life and their future integration into the working environment, whilst improving their productivity’.^6^

Students found their hosts to be welcoming and supportive of the placements. Whilst limitations of space were noted by both students and stakeholders, it did not appear to be a significant impediment to the completion and success of the research and health promotion activities.

Although most students agreed that the placements were helpful in training them to work with people of different backgrounds to their own, close to a third did not agree. This finding is important, as better understanding of the communities in which they may practice in future is an intended outcome of CBE. Medical professionals must be able to engage effectively with and be socially responsive to the needs of the highly diverse and multi-cultural societies of South Africa. Failure to achieve better sensitivity to issues of diversity reflects the difficulties of effective education in this regard.^25^

Students understood the critical role played by their host organisations in ensuring that communities had a stake in the projects. They strived to meet the stakeholders’ expectations and collaborate with them in the research and health promotion activities. Stakeholders described the experience as one of co-learning, allowing for a reciprocal transfer of knowledge as students provided significant insight into health issues and the health-seeking behaviour of the community. Similar collaborative approaches to community-based public health activities has been found to enhance understanding and integrate knowledge, allowing for improved health and well-being of the community.^26^ Students also provided a valuable service which, due to time and resource constraints, stakeholders themselves were unable to. This speaks to the mutually beneficial nature of collaborative partnerships as stakeholders gain access to complementary and otherwise limited resources and opportunities to build capacity.^27^

Students upheld principles identified by Wolff and Maurana^28^ as pivotal to effective partnerships, namely those of establishing trust with the community or community representatives; maintaining respect for their knowledge; shaping goals in conjunction with their needs; clearly specifying and acknowledging the various roles; providing feedback; building community capacity; and trying to establish community ownership of the activities and interventions. This latter aspect was identified by stakeholders to be a key challenge in respect of the sustainability of students’ interventions.

Both students and stakeholders considered the research and health promotion projects to be considerably beneficial to the communities. They increased awareness and knowledge of important health problems, dispelled myths surrounding many illnesses, and empowered communities to manage their health problems effectively. Community-based research and health promotion that values collaboration is increasingly being seen as a tool for community empowerment, as community members become vehicles of social change and improved community health outcomes.^27^

The limited time spent in the community mitigates against substantive research and sustainable health promotion interventions, which limit the benefits to communities. It is increasingly acknowledged that longer, integrated placements offer enhanced learning opportunities compared to traditional short rotations.^29^ Despite this, students and stakeholders alike reported that the projects had been useful in raising important health problems, addressing gaps in understanding and awareness of these problems, and effecting behaviour change in the community.

Many stakeholders have shared and disseminated students’ reports and tools with partners, and one stakeholder used student research as evidence in support of policy change at a national level. This demonstrates that student projects certainly have the potential to impact on a number of levels, and the literature highlights the potential for community-student partnerships to make meaningful changes to programmes, policy development and implementation.^26^

### Limitations of the study

The self-reported nature of the data is a potential reporting bias, especially in terms of recall bias. Many students declined participation or were unable to provide substantial responses because they felt that too much time had elapsed for them to remember their experiences clearly. Response rates, particularly amongst students from 2006 and stakeholders from 2006 and 2008, were particularly poor. There were also incomplete student responses to the survey questions. Due to the small sample size, it is difficult to generalise these findings to the broader population of students. Furthermore, the cross-sectional nature of this research precludes the authors from assessing how the CBE experiences may foster sustained changes to attitudes and practice behaviour in students, and the long-term impact of the research findings and health promotion interventions on the community and local healthcare services. Further longitudinal studies assessing objective outcomes would be particularly informative.

### Recommendations

It is recommended that there be regular formal evaluation on the impact and effectiveness of these placements as a benefit to the community and a formative influence on the students, to ensure the training of health professionals who are socially responsible and community-sensitive. Implementing the student and stakeholder recommendations will enable better realisation of the learning objectives and intended benefits of the placements.

## Conclusion

Community placements developed students’ skills and knowledge, particularly in research methods and health promotion; facilitated their engagement with patients and communities across cultural and social barriers; and promoted context-specific health awareness in helping communities to identify their health needs. Students conducted research into these issues and supported health interventions through the development of health promotion materials. The structure of the programme is particularly beneficial, as Seifer and colleagues (cited in Eckenfelds^30^) suggest that service learning ‘linked to specific course-based learning objectives has a greater impact on students than do electives’.

Despite the challenges, both students and stakeholders viewed the placements favourably and felt they achieved the learning outcomes and provided benefits for stakeholders and the communities. Whilst many students and some stakeholders felt these gains were relatively short term, useful and relevant material and resources were developed which stakeholders can share and use through further workshops and education sessions. Despite the limited duration of the placements, they offered valuable community-based learning experiences for the students and worthwhile benefits for the communities. Nevertheless, attention should be given to building on previous research and health promotion initiatives to improve sustainability. In addition, ways to improve the quality of student-community engagement should be considered to enhance the development of socially accountable values in students.
